# DNA Origami Nanomachines

**DOI:** 10.3390/molecules23071766

**Published:** 2018-07-18

**Authors:** Masayuki Endo, Hiroshi Sugiyama

**Affiliations:** Department of Chemistry, Graduate School of Science, and Institute for Integrated Cell-Material Sciences, Kyoto University, Sakyo-ku, Kyoto 606-8501, Japan

**Keywords:** DNA nanotechnology, DNA origami, DNA nanomachine, single-molecule analysis, high-speed AFM

## Abstract

DNA can assemble various molecules and nanomaterials in a programmed fashion and is a powerful tool in the nanotechnology and biology research fields. DNA also allows the construction of desired nanoscale structures via the design of DNA sequences. Structural nanotechnology, especially DNA origami, is widely used to design and create functionalized nanostructures and devices. In addition, DNA molecular machines have been created and are operated by specific DNA strands and external stimuli to perform linear, rotational, and reciprocating movements. Furthermore, complicated molecular systems have been created on DNA nanostructures by arranging multiple molecules and molecular machines precisely to mimic biological systems. Currently, DNA nanomachines, such as molecular motors, are operated on DNA nanostructures. Dynamic DNA nanostructures that have a mechanically controllable system have also been developed. In this review, we describe recent research on new DNA nanomachines and nanosystems that were built on designed DNA nanostructures.

## 1. Introduction

DNA nanotechnology is growing rapidly and is widely accepted as a tool in multidisciplinary research fields. DNA can control the formation of double-stranded DNA (dsDNA) through selective sequence-dependent base pairing, and the expected structures are formed based on a periodic double-helical geometry. This technology allows the construction of various self-assembled structures that are used for the placement and arrangement of functional molecules and nanomaterials, to produce complex molecular devices. DNA origami—i.e., a programmed DNA assembly system based on the well-established DNA nanotechnology—enables the design of two- and three-dimensional (2D and 3D) nanostructures with a wide variety of shapes and a defined size [1,2]. In addition to structure design, DNA is used for the generation of molecular machines that have a controllable molecular system that enables complicated movements. The double-helical structure is formed via hydrogen bonding of base pairs, so that the dissociation and association of complementary DNA strands can be controlled reversibly by heating and cooling, respectively. This means that the molecular assembly of DNA strands can be manipulated via the dynamic control of the association and dissociation of the DNA strands. As the thermodynamic parameters of the base pairing between DNA strands are predetermined, suitable DNA sequences can be designed to generate molecular switches for the control of the movement of DNA nanomachines [3]. The DNA molecular machines have been combined with the DNA nanostructures, and mechanical nanodevices are currently being created with nanoscale precision; the mechanical parts of these nanodevices are operated by specific molecules, metal ions, and external stimuli, such as light, pH, and temperature [4]. In addition, as the DNA nanostructure can be customized using functional molecules and nanomachines, there is a great advantage to create a device in which each function is combined as a module.

In this article, we review the recent progress in the research on DNA nanomachines and nanosystems constructed in the designed DNA nanostructures. We also describe the applications of DNA origami nanomachines to optical and biological devices.

## 2. Controllable DNA Nanomachines and Designable DNA Nanostructures

### 2.1. DNA Nanomachines

**Figure 1:**
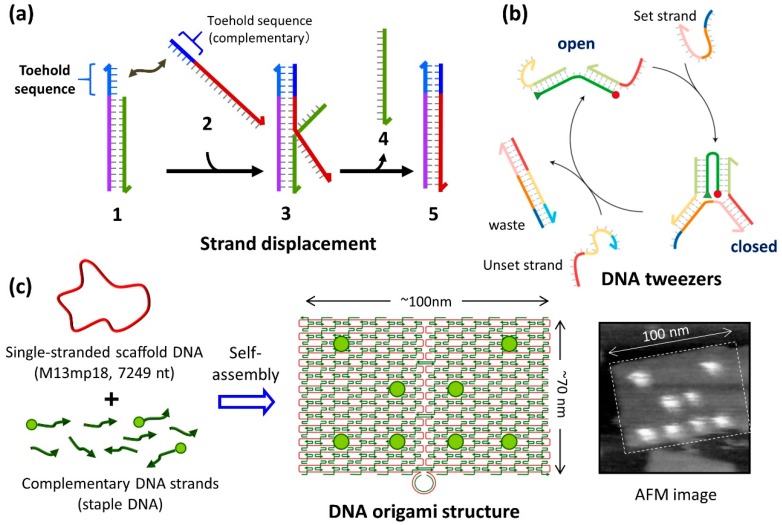
Operation of DNA machines and creation of nanostructures by DNA origami method. (**a**) DNA strand displacement reaction via toehold sequence used for operation of DNA nanomachine. (**b**) DNA tweezers using strand displacement reaction [5]. Set strand controls open to close state of the green DNA strand and unset strand removes the set strand to make open state. (**c**) The preparation of nanometer scale structure using DNA origami method. Long ssDNA (M13mp18) and short complementary DNA strands (staple DNA) are self-assembled by annealing [1]. When the target molecules (green circle) are bound to the staple DNA, the molecules can be placed at the predesigned positions on the DNA origami structure.

DNA nanomachines are inspired by the biological molecular machines that can be observed in living systems. Several nanomachines have been created to realize rotational, reciprocating, and walking motions [3]. DNA molecular machines are mainly operated by the addition and removal of specific DNA strands, to induce the hybridization and dehybridization of DNA strands. In addition to hydrogen bonding, base-stacking plays a major role on double-stranded DNA hybridization. In particular, DNA strands with an additional DNA sequence called “toehold” are used to accomplish diverse and complex movements. The addition of a complementary strand containing a toehold part allows the isothermal induction of DNA strand exchange from the preformed dsDNA (strand displacement reaction) (Figure 1a). Therefore, the DNA strands can be replaced in the thermodynamically stable direction using the difference in stabilization energy for the hybridization of DNA strands. Using this method, DNA tweezers that can switch and open to a closed structure reversibly were created by controlling the binding and dissociation of two DNA strands (set and unset strand) (Figure 1b) [5]. DNA molecular systems, such as DNA walkers (which are fully controlled by multiple strand displacements and DNA motors that move forward autonomously via a nicking enzyme), have been constructed to achieve directional movement [3]. As the DNA hybridization/dehybridization of toehold-containing strands are used for the operation of the nanomachines, the operation speed is affected by the length and sequence (C/G and A/T content) of the toehold part [6]. Therefore, the order of the operation speed depends on the kinetics of the hybridization and dehybridization of DNA strands.

In addition, nucleic acid switches have been developed to control the association and dissociation of DNA strands [7]. The metal ions and pH conditions induce the switching, for example, K^+^ induced G-quadruplex formation, acidic condition induced triple helix formation, and metal-ion-induced base complex formation using T-Hg^2+^-T and C-Ag^+^-C. Photochemical switches such as DNA strands containing photoisomerization units such as azobenzene derivatives are available for the reversible control of hybridization and dehybridization of the DNA strands [8,9]. These switches are also available for regulation of the movement of nanomachines.

### 2.2. Construction of DNA Nanostructures

DNA is also an excellent material for the construction of accurate nanoscale structures. To create nanoscale structures via duplex formation, various methods have been developed that are based on the complementarity of the DNA sequence and the periodicity of the DNA double-helical structure. DNA origami, which allows the design and creation of various 2D nanostructures, was developed in the past decade and has been widely investigated to create novel functionalities [10]. DNA origami was initially developed for the construction of planar nanostructures with a defined size and shape (~100 nm) that are formed via the self-assembly of sequence-designed DNA molecules [1,2,11]. In this method, long single-stranded DNA (ssDNA) (M13mp18) and complementary DNA (staple DNA) strands with sequences that are designed according to the target structures are mixed together, and the mixture is heated and then cooled slowly (annealing), to form the predesigned structure via self-assembly (Figure 1c). The structures are confirmed using atomic force microscopy (AFM). In addition, the strategies for design and construction of the three-dimensional (3D) origami structures were crated and computer-assisted design software caDNAno was developed [2,11,12]. Nowadays 3D structures are routinely confirmed with transmission electron microscope (TEM). As different staple DNAs are used at all the positions in the formed DNA origami structure, extra DNA strands can be placed at a desired position on the planar structure. Because various biomolecules, functional molecules, and nanomaterials are attached to the synthetic DNA strands, these molecules and materials can be placed into the DNA nanostructure in a position-specific manner, to create new functions.

We have developed a method for directly observing the behavior of enzymes and DNA structural changes using high-speed AFM, and controlling the reaction at a molecular resolution by fixing substrate DNA strands on the DNA origami [13,14]. In addition to its convenience for the purpose of single-molecule observation, various manipulations of the molecules can be performed in the nanoscale space constructed in the DNA origami structure. We used this method to visualize directly the movements of biomolecules and synthetic molecules in a defined DNA origami nanostructure using high-speed AFM, for the characterization of the properties of target molecules at the single-molecule level.

## 3. Direct Observation of a Mobile DNA Nanomachine on the DNA Origami Surface

### 3.1. DNA Molecular Machine on the DNA Nanostructure

For complex movements of DNA nanomachines, a controllable molecular system in which the nanomachines are operated by specific DNA strands in a programmed fashion is needed. Seeman and coworkers performed pioneering studies of combining molecular machines with DNA nanostructures. Using the conformational change of dsDNA called B–Z transition, in which the dsDNA conformation changes from a right-handed (B-form DNA) to a left-handed (Z-form DNA) conformation, a reciprocating motion of the DNA nanostructure was observed [15]. In addition, those authors developed molecular machines that are capable of rotating 180° at the ends of two adjacent dsDNAs, termed PX-JX_2_ devices, by hybridization and removal of DNA strands [16]. They also successfully captured triangular DNA nanostructures using the sequence specificity of four single-stranded ends by introducing two devices on DNA origami and rotating each triangle [17]. Using this method, four types of PX-JX_2_ patterns could be operated by specific DNA strands, and four different types of nanostructures were captured.

**Figure 2:**
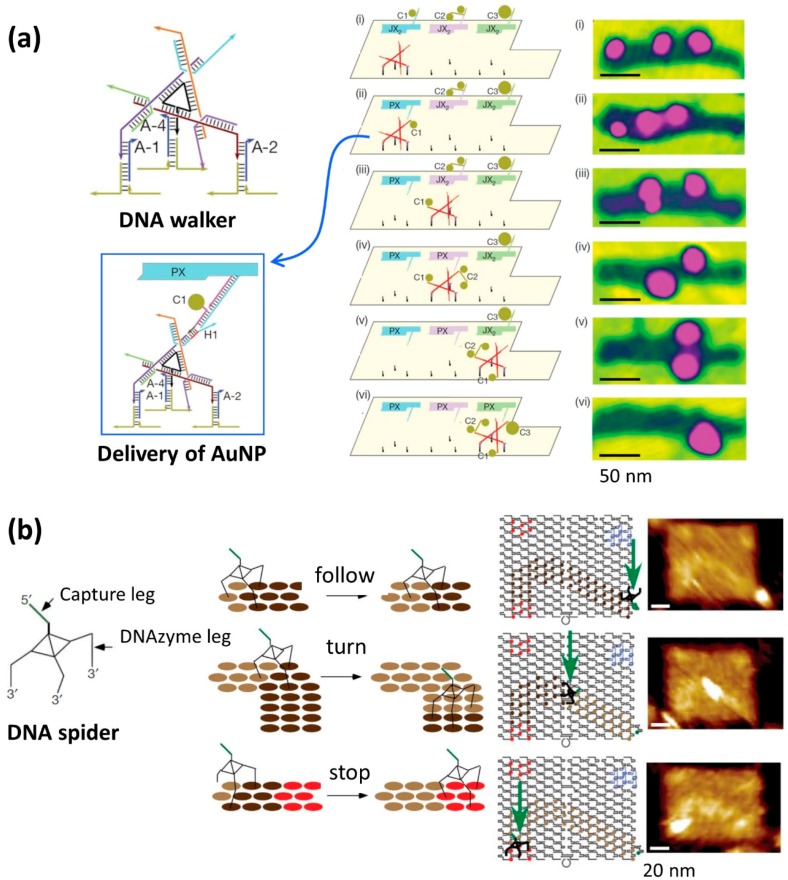
Assembly line with a DNA walker capturing gold particles and DNA spider molecule walking in a track on a DNA origami. (**a**) The DNA walker binds to the DNA strand on the DNA origami with three legs, and gold particles (AuNPs) are collected with three hands [18]. The DNA walker stops at three places on DNA origami and receives AuNPs (C1, C2, C3) to be transferred by rotating PX-JX_2_ DNA devices. Multiple operation on DNA origami and corresponding AFM image. (**b**) The DNA spider binds onto the DNA origami using three legs hybridized to ssDNAs (cleavage site is RNA) in the track. DNAzyme for cleavage of RNA site in the ssDNA is introduced to three legs [19]. DNA strands in the track before and after cutting (brown and light brown circles) and stopping DNA strands (red circles). The path for walking with instruction (start, follow, turn, and stop) can be programmed on the DNA origami. AFM image of DNA spider molecule walking on the DNA origami track. Start (top), walking (middle), and stop (bottom).

Furthermore, Seeman and coworkers created an assembly line in which a DNA walker was operated on the DNA origami and captured multiple gold nanoparticles (AuNPs) (Figure 2a) [18]. The authors arranged three PX-JX_2_ devices and operated a DNA walker that moved on a predesigned track (route). All device movements and DNA walker movements were controlled by specific DNA strands. Various AuNPs with different sizes were placed on the PX-JX_2_ devices and were transferred to the DNA walker by a rotating motion at the specific positions. These results show that the DNA walker moved under the control of three points on the track in one direction, that the delivery of AuNPs was controlled by the PX-JX_2_ devices, and that the AuNPs were transferred to the DNA walker. After all of these procedures were performed, DNA walkers with three types of AuNPs bound to them were finally obtained with a yield of 43%. As the PX-JX_2_ device can control the ON–OFF switching of the transfer of AuNPs using specific DNA strands, the product can be obtained in high yield (>90%), and the error rate is virtually suppressed (1%). In addition, eight patterns of capture of AuNPs onto the DNA walker were achieved using three PX-JX_2_ devices by the ON–OFF switching operation of these devices.

Yan and coworkers demonstrated the walking of DNA molecular machines called “DNA spiders” on the various path patterns constructed on the DNA origami (Figure 2b) [19]. DNA spiders consisted of three DNA strand legs and one capture DNA strand (capture leg). The three legs of the DNA spiders contained a DNA enzyme (DNAzyme leg) that could hydrolyze RNA. Single-stranded DNAs containing a cleavable RNA site were arranged on the DNA origami as a track for the walking of the DNA spider. The DNA spider was immobilized at a specific position on the DNA origami using a trapping DNA strand, followed by dissociation and initiation of the walking on the track. The DNA spider bound to the ssDNAs on DNA origami, cleaved the RNA site in the ssDNAs using its DNAzyme moiety, and walked autonomously. The DNA spider moved forward along the predetermined track and finally stopped at the specific area on DNA origami where ssDNAs did not have an RNA site for cleavage. All of these processes, including the initiation, walking on the track, and stopping, were controlled in a programmed way. In addition, by measuring the position of the DNA spider on the DNA origami using super-resolution microscopy, it was found that the spider moved at a speed of 3 nm/min.

### 3.2. Walking of the DNA Motor on Predesigned Tracks

**Figure 3:**
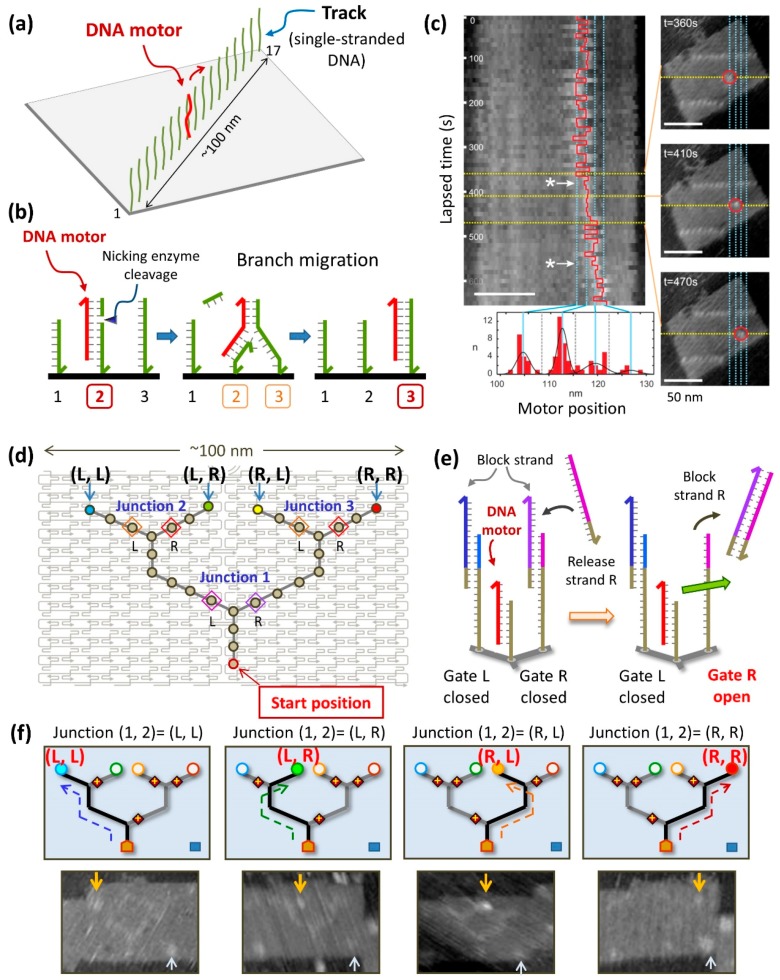
A DNA motor system constructed in the DNA origami structure [20]. (**a**) A track consisting of 17 ssNAs (green) constructed on a DNA origami structure. The DNA motor strand (red) moves on the track in one direction using enzymatic reaction. (**b**) Mechanism of DNA motor system using branch migration. After cleavage of nicking enzyme, the DNA motor hybridized to the ssDNA (green) in the track moves to the adjacent DNA strand with the same sequence via intermediate state (branch migration). (**c**) Single-molecule visualization of the DNA motor using high-speed AFM and its analysis. The DNA motor moved stepwise along the ssDNAs in the track, and the intermediate state of the branch migration could also be visualized. (**d**) A DNA motor system using a branched track and controllable gates [21]. A track consisting of ssDNAs having three branch points (junctions) and four end points constructed on the DNA origami structure. The DNA motor moves along the ssDNAs in the branched track from the start position. (**e**) The control of opening the gates constructed on both sides of the branch point by strand displacement. (**f**) The branched track controlled by multiple gates and the programmed movement of the DNA motor by following the instructions. AFM images of the DNA motor movement directed by the designed instruction.

We also created a DNA motor system on the DNA origami that had a track (~100 nm) consisting of 17 ssDNAs, for the observation of the movement of a complementary DNA strand (DNA motor). We expected that the DNA motor would move continuously in one direction (Figure 3a) [20]. The principle of the operation of the DNA motor is as follows. As shown in Figure 3b, ssDNAs are incorporated onto the DNA origami, the DNA motor strand is hybridized at that site, and the nicking enzyme Nt.BbvCI selectively cleaves the duplex of DNA motor/DNA substrate strand in the track. Subsequently, the shortened cleaved DNA strand dissociates, and the motor strand moves forward to the adjacent DNA strand with the same sequence. This is called branching migration. As ssDNAs with the same sequence are arranged at equal intervals on the track, enzymatic cleavage of the DNA strands and branch migration of the DNA motor proceed autonomously. After introducing the DNA motor at the end of the track, the position of the DNA motor was observed by AFM. The DNA motor moved in one direction after the reaction. The movement of the DNA motor on the track was directly observed during AFM scanning (Figure 3c). In addition, the analysis of the movement of the DNA motor revealed that the intermediate state of the DNA motor during branch migration could be visualized by high-speed AFM and that the DNA motor moved stepwise along ssDNAs in the track.

**Figure 4:**
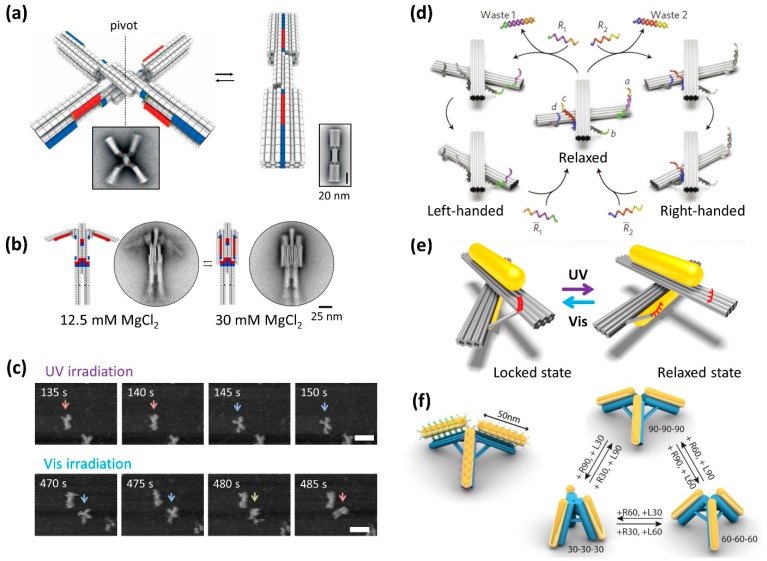
Reconfigurable DNA origami structures that change their conformation in response to physical stimuli and DNA strands. (**a**) DNA dynamic nanodevice that responses to temperature and salt concentration. Two domains can rotate around the center axis. The nanodevice reversibly opens and closes in response to temperature [24]. (**b**) A molecular robot that opens and closes the arms in response to salt concentration. (**c**) HS-AFM images of conformational changes of the DNA nanodevice with photoswitching strands and UV and Vis irradiation [25]. (**d**) Reconfigurable DNA nanostructure in left-handed and right-handed locked states controlled by strand displacement with toehold-containing DNA strands [26]. (**e**) Plasmonic nanostructure with two gold nanorods (AuNR), and locked and relaxed state can be controlled with photo-responsive DNA strands and UV/Vis irradiation [27]. In response to light, a locked state and a relaxed state occur, and spectroscopically read out according to the plasmon interaction between the AuNRs. (**f**) Reconfigurable DNA origami tripod with AuNRs. Releasing strands (R) and locking strands (L) are employed to stepwise manipulation of the angle between the DNA arms [28].

Furthermore, to control the movement of molecules with nanoscale precision, we created a DNA motor system using a complicated path constructed on the DNA origami (Figure 4) [21]. We created a branched track on the DNA origami that contained gates on both sides of the three branching points (junctions), to control the walking direction of the DNA motor. The gates were closed beforehand using blocking strands, and DNA motors passed through only when the strands blocking the gates were removed. The position of the DNA motor after enzymatic reaction was examined by AFM analysis and fluorescence quenching. The DNA motor moved forward in the direction of the opened gates and finally reached the four designated end points. We successfully developed this DNA motor system by constructing a complicated branched path on the DNA origami and enabled the nanoscale control of the direction of the movement of a DNA motor in a programmed fashion.

We also constructed a light-driven DNA motor system using photochemical reactions [22]. The movement of the DNA motor driven by light was examined on the DNA origami structure. Four DNA strands containing a disulfide bond among the first three strands were placed on the DNA origami, which worked as a track. A pyrene-attached DNA motor was introduced and hybridized at the first position [23]. Electron transfer from pyrene moieties using UV light irradiation induced the cleavage of disulfide bonds, leading to continuous movement of the DNA motor until it reached the last ssDNA along the track. According to the irradiation time, the position of the DNA motor was determined by the positions of the four ssDNAs on the DNA origami, and the reaction speed of each step was estimated as 0.95 × 10^−2^ to 1.3 × 10^−2^ s^−1^ from the distribution of the positions of the DNA motor. In addition, the movement of the DNA motor could be observed directly in real time using high-speed AFM under UV irradiation. 

## 4. Mobile DNA Origami Structures

### 4.1. Controllable DNA Origami Nanomachine

One of the objectives of DNA nanotechnology is the creation of molecular machines, molecular robots, and mechanical devices [29]. Robust mechanical devices were designed and constructed using the relatively rigid 3D origami structures [2]. Three-dimensional DNA origami that change structure in response to salt concentration and temperature have also been developed (Figure 4a) [24]. The system uses the π–π stacking interaction and shape fitting that occur between the base pairs of dsDNA terminals. In this design, a structure consisting of two pluggable rods can be rotated at the center, which can lead to a change between the opened and closed forms. The X-shaped structure (open form) was closed by a shape fitted according to the salt concentration and changed to the rod shape (closed form). Using fluorescence changes to detect the open/close behavior by controlling the temperature, the structure worked without breaking even when the opening and closing of the structure was repeated more than 1000 times. This showed excellent performance as a component of the molecular machine. This component was further assembled to construct a movable lattice structure at the micrometer scale. In addition, a nanoscale robot that can open and close arms depending on the salt concentration was constructed by incorporating movable parts (Figure 4b).

This switchable structure was further modified with photoresponsive molecules to control the open/closed conformations by photoirradiation [25]. Using photoswitching DNA strands containing azobenzene moieties [9], a photoresponsive structure was created in which the open and closed conformations could be distinguished by gel electrophoresis and AFM imaging after respective irradiation with UV and visible light. Moreover, these reversible changes in shape during photoirradiation were directly visualized by high-speed AFM. Under UV irradiation, the closed nanostructure opened during fluctuation; subsequently, the opened nanostructure closed under Vis irradiation (Figure 4c). These results indicate that the opening and closing of the nanostructure can be reversibly controlled by merely switching the light source. Moreover, four photoswitchable nanostructures were assembled into a tetramer. A scissor-actuator-like higher-order object in which the configurations could be controlled by the open and closed switching was induced by UV and Vis light irradiation. As seen here, the capability of switching was preserved even while the structures were attached onto surfaces, which may offer interesting possibilities for constructing light-responsive electronic or photonic devices using DNA origami switches.

### 4.2. Controllable DNA Origami Optical Device

One of the objectives of the development of controllable DNA nanostructures is the generation of optical devices. Synthetic molecular machines typically operate at the nanometer scale or below. Using 10- to 100-nm-sized DNA nanostructures and molecular machines, the controlled operation of individual molecular machines with a larger dimension should be achieved and have many practical applications. Two bar-shaped recofigurable DNA nanostructures connected at the center as a pivot were constructed and their direction of rotation and left-handed and right-handed locked states were controlled by strand displacement with toehold-containing DNA strands (Figure 4d) [26]. We created a light-driven plasmonic nanosystem that has photoswitches attached to the host nanostructure and exhibits a reversible chiroptical function with large-amplitude modulation [28]. The recofigurable DNA nanostructures were functionalized with photoswitching DNA strands [9] and its locked and relaxed states were controlled by visible and UV light irradiation, respectively. Two gold nanorods (AuNRs) were assembled onto a reconfigurable DNA origami scaffold, to create the plasmonic nanostructure (Figure 4e) [27]. In the locked state, the expected peaks were observed in the circular dichroism (CD) spectra, while no peak was observed in the relaxed state because of the random positioning of the two AuNRs. Reaction rates for the unlocking and locking were obtained as 5.0 × 10^−3^ and 1.3 × 10^−2^ s^−1^, respectively. Reversible switching of the relaxed and locked states of the plasmonic nanostructure was achieved by alternating UV with visible light irradiation, respectively. An all-optically controlled plasmonic nanosystem was constructed on a designed DNA nanostructure, which can be read out using optical spectroscopy. Light can reversibly “write” and “erase” the conformation states of the nanostructure. This plasmonic nanosystem provides the unique features of optical addressability, reversibility, and modulability, which are crucial for the development of all-optical molecular devices with desired functionalities.

Also using a reconfigurable DNA origami tripod, three AuNRs were attached to structure and the angle and distance between AuNRs were precisely controlled by toehold-mediated strand displacement (Figure 4f) [28]. Reversible conformational change of three different structures was detected by shift of the plasmonic resonance peak and characterized by dark-field scattering spectra. Using reconfigurable DNA origami, various 3D plasmonic nanostructures can be constructed and used to study the plasmonic resonance of AuNRs, and their optical properties can be controlled by strand displacement and photoreaction.

### 4.3. Dynamic Rotary Motor System

**Figure 5:**
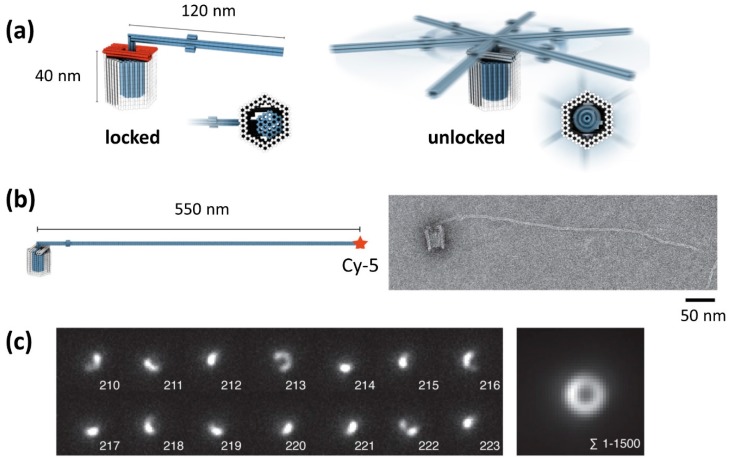
DNA-based rotary apparatus [32]. (**a**) Design of assembled rotor apparatus with closed brackets (red) and a locked rotor (blue) (left). Assembled rotary motor apparatus with a mobile rotor (right). (**b**) Rotary apparatus with a 550 nm crank lever for observation of rotary movement. TEM image of the construct. (**c**) Observation of rotary movement of single apparatus acquired by TIRF microscopy (left) and sum over all 1500 images (right).

The biological rotary motors such as bacterial flagellar motor [30] and F_1_F_0_–adenosine triphosphate (ATP) synthase [31] play important role in the living system. For mimicking the native rotary motors, the rotary apparatus was constructed from three different DNA origami components; a rotor unit and two clamp elements that form an axle bearing (Figure 5a) [32]. The architecture had a bearing cavity and a cylindrical envelope of the rotor unit. Three units were assembled in a stepwise fashion using shape-complementary of the components. Finally, a mechanically interlocked architecture was constructed by closing the top (Figure 5b).

For observation of the rotary mechanism, the crank lever of the rotor unit was extended to be a length of 550 nm and fluorescence dyes were attached to the end. Using this apparatus, the rotary movements were observed by total internal reflection fluorescence (TIRF) microscopy. Arc-like optical signatures were frequently observed in individual frames (Figure 5c, left). By summing up 1500 frames, a donut-like optical signature with a diameter of ~1 μm was observed, which corresponds to the rotational diameter of the crank lever (Figure 5c, right). The rotation behavior can be changed by adjusting the solution conditions and the bearing variant in the apparatus. The rotor can dwell in docking sites or randomly rotate around the central axis of the bearing. These results suggests that mobility of the rotor, the dwell positions and time can be controlled through rational design. This prototype of the rotary apparatus can be a platform for creating synthetic rotary motors.

## 5. Mechanical Detection of the Interaction of Molecules

### 5.1. DNA Origami Channel with Gating

**Figure 6:**
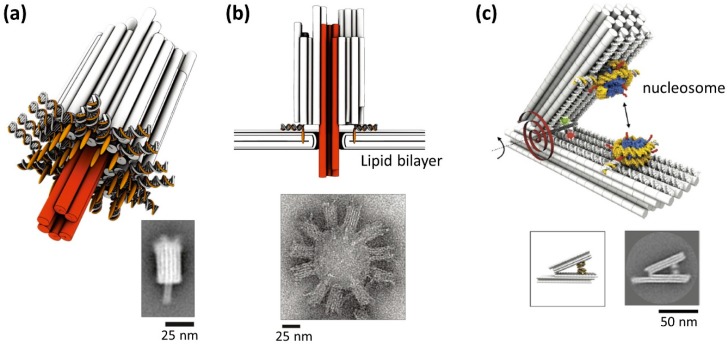
(**a**) DNA origami channel [33]. Tubular pores (red), main body (gray), cholesterol moieties (orange) at the bottom of the main body and TEM image. (**b**) The DNA channel is bound to the lipid bilayer membrane via cholesterol, and the pore domain penetrates the lipid bilayer membrane. TEM image of origami channels bound to a liposome. (**c**) Measurement of nucleosome-nucleosome interaction using reconfigurable tweezer-shaped DNA origami and TEM image [34].

Robust 3D DNA origami can be used for construction of artificial channels. Channels are formed in the cell membrane to transfer the specific molecules and ions with a controlled gate system. DNA origami transmembrane channels were created and incorporated in a lipid bilayer [33]. This architecture consists of a robust 3D body with hollow inside and a short tubular pore to penetrate a lipid membrane (Figure 6a). To tightly attach to the lipid bilayer surface, multiple cholesterol molecules were incorporated to the bottom of the main body of the channel structure. Using single-channel electrophysiological measurements, response of the origami channel showed similar properties compared to the natural ion channels in terms of conductance and channel gating. Using this channel, different lengths of single-stranded DNAs with a stopper such as a stem and G-quadruplex were discriminated by electrophysiological measurements. The results show that the origami channels can be further used to identify target molecules by detecting single-molecule translocation.

### 5.2. Detection of Interaction of Molecules Using Dynamic DNA Nanostructures

Actuators associated with molecular detection were created using DNA origami. A scissor-type mechanical DNA origami responding to specific molecules was created [35]. When the nanostructure captured the specific molecule, two arms closed to pinch the molecules; this was used to detect single target molecules. Also a rhombus-shaped DNA origami nanoactuator which changed its conformation responding to the length of adjustable strands using specific DNA strands [36]. This nanoactuator was further used as a nanosensor that responded to ions, restriction enzymes, or specific RNA strand.

The characterization of the molecular interaction is one of the utilizations of the mechanical DNA nanostructures. For understanding higher-order chromatin structures that regulate genome structures, the energy landscape for nucleosome association is important. Using a tweezer-shaped DNA origami for integrating two nucleosomes, interaction force between nucleosomes was measured (Figure 6b) [34]. This reconfigurable DNA origami can work for force spectrometer to measure in sub-nanometer resolution. The distance between two nucleosomes was measured by electron microscopy imaging. From these measurements, relative nucleosome orientation did not affect the interaction of the nucleosomes. However, acetylation of histone H4 and removal of histone tails weakened the interaction drastically. This force spectroscopy can be a powerful tool for the studying the physical properties of the molecular interaction in high-resolution.

## 6. DNA Nanorobots for Biological Applications

### 6.1. Nanorobot with Dynamic Mechanism

**Figure 7:**
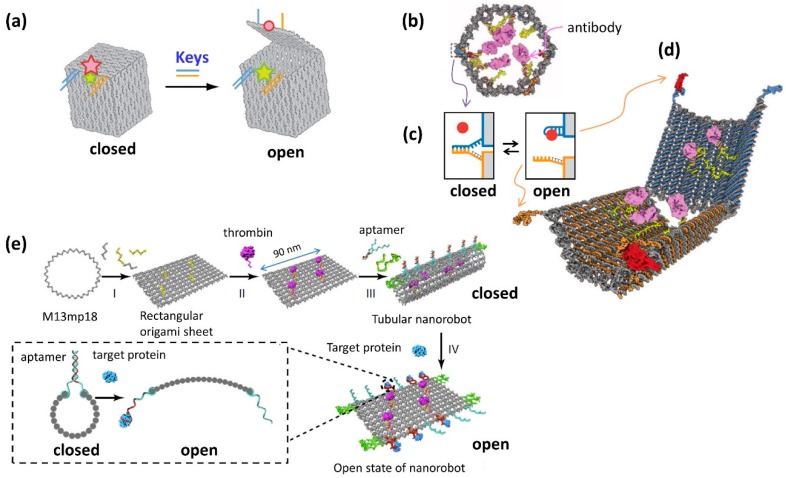
DNA nanorobots with open/close switch for biological applications. (**a**) Controlled opening of the box lid using toehold containing DNA strands (keys) [37]. (**b**) DNA nanorobot that recognizes cells and activating signaling pathway in the cell [39]. Nanorobot in a closed state. Antibodies are attached inside the barrel-shaped structure and are closed by DNA strands that used as “locks” (dashed rectangle). (**c**) Mechanism of opening the structure by use of “key”. The target molecule (red circle) binds to the blue DNA strand (aptamer DNA), and the initial dsDNA dissociates. (**d**) Nanorobot in an opened state. Internal antibodies bind to cell-specific antigens. The nanorobot opens with two kinds of target molecules, so that only when two molecules exist, it binds to the cell surface. Cells can be Identified in a logic gated way. (**e**) DNA nanorobot targeting specific tumor [29]. Thrombin is attached to the sheet, and the structure is closed in a tubular shape by aptamers. When a tumor associated target protein is attached to the aptamers, the tubular nanorobot opens to expose the thrombin, which performs blood coagulation at the tumor site.

One of the goals of the development of DNA molecular machines is their application to the control of cellular functions. DNA nanostructures are also used for the container of the molecules, which have dynamic open/close system to release or expose target molecules. The first example of the dynamic nanostructure having open/close system is a DNA box, whose lid opening is controlled by strand displacement with toehold containing DNAs (Figure 7a) [37]. The octahedral structure with a photoresponsive open/close system was constructed to include and release the gold nanoparticle [38]. A DNA nanomachine called a “DNA nanorobot” can recognize target biomolecules on the cell surface, which induces a 3D structure change and selectively controls cellular functions [39]. Nanorobots with a hexagonal barrel shape were opened into two domains, and one end of the structure had hinges, to allow the opening and closing of the structure (Figure 7b). When the target molecule bound to the connecting dsDNAs (locks) in the closed structure, the structure was opened by dissociation of the connecting dsDNA (Figure 7c). The barrel structure was designed to open by recognition and binding to the target molecules on the cell surface (Figure 7d). When the barrel structure was designed to open with two types of target molecules, the barrel could be opened using only two types of target molecules that exist on the cell surface. A specific antibody was also incorporated into the structure. The opening of the tubular structure by recognition of the target biomolecules on the cell surface led to the binding of this antibody to the receptor on the cell surface and subsequently activated a signaling pathway to induce cell death or cell division, as instructed. Using this system, cancer cells can be killed based on the recognition of cellular markers. Nanorobots may represent an innovative type of medical molecular robot that can be programmed using various biomolecules.

### 6.2. Nanorobot Targeting Tumor In Vivo

DNA nanorobots with dynamic mechanisms also have great potential for an intelligent drug delivery system that responds to target molecules [40]. DNA origami structure with a programmed system was designed, which can respond to specific molecules and designed to present molecules effective for tumor (Figure 7e) [41]. Nanorobot was functionalized with a DNA aptamer that binds to a protein specifically expressed on tumor-associated endothelial cells on the outside, and a blood coagulation protease thrombin inside the nanorobot. The aptamer that responds to the target protein of the tumor cell functions as a lock for the DNA nanorobot to open mechanically. When the nanorobot opens, the thrombin introduced inside is exposed and promotes blood clotting at the tumor site. Using a mouse model with tumor, DNA nanorobot introduced into the blood deliver thrombin specifically to the tumor-associated blood vessel, induce tumor necrosis and inhibit tumor growth. In addition, the nanorobot was immunologically inactive in vivo. This demonstrates that the strategy to use target selective DNA nanorobots is promising for accurate drug delivery and the cancer therapy.

## 7. Summary and Perspectives

The progress of DNA nanotechnology has allowed the design and construction of various 2D and 3D DNA nanostructures. DNA origami technology in particular has enabled the precise arrangement and manipulation of biomolecules and functional molecules, and nanoscale spaces for reaction and observation have been constructed. In this research, the dynamic manipulation of molecules has been combined with the DNA origami structure, and molecular nanomachines have been constructed and operated. In addition, techniques have been developed for setting a route on a DNA origami structure, to control the movement of a DNA nanomachine. Using a gate with a traveling direction that can be determined by a complicated branching path, one molecule can be moved to a specific location. In addition, a technique for controlling the motion of single molecules based on a photochemical reaction has been developed. Furthermore, dynamic DNA origami devices such as reciprocal and rotary nanodevices have been developed, and the movements are controlled by incorporated mechanical switches that are responsive to target molecules and external stimuli. More advanced nanorobot, which can control target cellular functions and tumor treatment, have been developed. This reversible open and closed system is used to initialize the configuration of the nanostructures and to change them into specific shapes repeatedly by mere irradiation with a specific wavelength of light. The photonic modification of DNA-based nanomachines can be used in biological applications, such as cargo transport and the manual change of the configuration of biomolecules in mesoscopic systems. Furthermore, these nano-sized switchable molecular devices could be used as reconfigurable nanomaterials and may become useful tools that can be used to regulate biochemical reactions. These systems enable the transport and release of target molecules in a spatiotemporal and autonomous way. A technology aimed at regulating the dynamic movement of molecules with nanoscale precision is still under development, and there is a possibility of developing an intelligent “molecular robot” that processes environmental information autonomously into computation and actuation.
